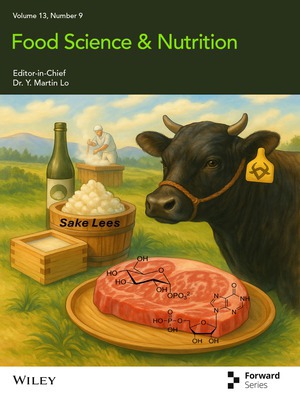# Cover Image

**DOI:** 10.1002/fsn3.70921

**Published:** 2025-09-18

**Authors:** Hitomi Shikano, Kazuki Komatsu, Fumiya Koga, Meguru Hara, Kazuaki Yoshinaga, Naoto Ishikawa, Shu Taira

## Abstract

The cover image is based on the article *Taste Enhancement in Japanese Black Wagyu Beef Fed With Sake Lees: Insights From Metabolomic and Sensory Evaluations* by Hitomi Shikano et al., https://doi.org/10.1002/fsn3.70839.